# Prognostic Role of Platelet-to-Lymphocyte Ratio in Patients With Bladder Cancer: A Meta-Analysis

**DOI:** 10.3389/fonc.2019.00757

**Published:** 2019-08-14

**Authors:** Xingmu Wang, Xiaoyan Ni, Guiliang Tang

**Affiliations:** ^1^Center of Clinical Laboratory, Shaoxing People's Hospital, Shaoxing Hospital of Zhejiang University, Shaoxing, China; ^2^Department of Urology, Shaoxing People's Hospital, Shaoxing Hospital of Zhejiang University, Shaoxing, China

**Keywords:** meta-analysis, PLR, prognosis, bladder cancer, biomarker

## Abstract

**Background:** Many studies have been reported that platelet-to-lymphocyte ratio (PLR) may be associated with the prognosis of bladder cancer, but the results are inconsistent. Therefore, we performed a meta-analysis to evaluate the effect of pretreatment PLR on the prognosis of bladder cancer.

**Methods:** The databases PubMed, Embase, Cochrane Library, and Web of Science were searched. Pooled hazard ratios (HRs) and 95% confidence intervals (CIs) were used to analyze the relationship between PLR and prognosis. Pooled odds ratios (ORs) and 95% CIs were used to analyze the relationship between PLR and clinicopathological features. Publication bias was estimated using Begg's funnel plot asymmetry tests.

**Results:** A total of 8 studies comprising 3,303 patients were included in this meta-analysis. An elevated PLR was significantly associated with poorer overall survival (OS) (HR = 1.26, 95% CI = 1.03–1.54, *p* = 0.026), but not with cancer-specific survival (CSS) (HR = 1.15, 95% CI = 0.95–1.38, *p* = 0.149), or recurrence-free survival (RFS) (HR = 1.72, 95% CI = 0.79–3.75, *p* = 0.175). In addition, high PLR was correlated with age ≥ 65 years (OR = 1.82, 95% CI = 1.24–2.67, *p* = 0.002), whereas was not significantly correlated with sex, tumor grade, tumor stage, distant metastasis, or tumor size.

**Conclusions:** The pretreatment PLR could serve as a predicative biomarker of poor prognosis for patients with bladder cancer.

## Introduction

Bladder cancer is the 10th most common cancer worldwide, with an estimated 549,000 new cases and 200,000 deaths in 2018 (1). Approximately 75% of all bladder cancer cases occur in men and incidence rates varies largely around the world (1). Basically, bladder carcinomas are classified as non-muscle invasive bladder cancer (NMIBC) (Ta/T1) and muscle-invasive bladder cancer (MIBC) (T2–T4). About 75% of patients have NMIBC and 25% have MIBC or metastatic disease (2). Despite aggressive surgical treatment and improvements in therapeutic approaches for bladder cancer, survival outcome has not substantially improved, with high recurrence and mortality (3). Prognostication is essential for treatment decision making (4). Therefore, seeking a novel and effective prognostic biomarker is important for the improvement of survival outcomes.

Host inflammatory responses can greatly affect tumor development and progression (5). The systemic inflammatory status could be reflected by many blood biomarkers including C-reactive protein (CRP), neutrophil-to-lymphocyte ratio (NLR) (6), platelet-to-lymphocyte ratio (PLR) (7, 8), and lymphocyte-to-monocyte ratio (LMR) (9). Various serum and tissue biomarkers reflecting systemic inflammatory response show reliable prognostic value in cancer (6). PLR is calculated as platelet counts divided by lymphocyte counts. Previous studies have reported the prognostic value of PLR in various solid tumors, such as hepatocellular carcinoma (10), breast cancer (11), colorectal cancer (12), prostate cancer (13), and non-small cell lung cancer (14). Recently, several retrospective studies have evaluated the impact of PLR on the prognosis of bladder cancer patients (15–19). However, based on their findings, the current view of the prognostic role of PLR in bladder cancer is not yet clear. Therefore, we performed a meta-analysis to evaluate the effect of pretreatment PLR on the prognosis of bladder cancer.

## Materials and Methods

### Search Strategies

This meta-analysis was carried out according to Preferred Reporting Items for Systematic Reviews and Meta-Analyses (PRISMA) Statement (20). The databases PubMed, Embase, Cochrane Library, and Web of Science were searched to March 2019. The citation lists of included studies were also examined. Search terms included “bladder cancer” or “bladder carcinoma” or “bladder neoplasm” or “bladder tumor,” and “platelet to lymphocyte ratio,” or “PLR,” or “platelet-lymphocyte ratio.” Ethical approval was waived because this was a meta-analytic study and we just collected the data from available publications.

### Inclusion and Exclusion Criteria

The inclusion criteria were as follows: (1) bladder cancer was diagnosed from pathological examination; (2) a dichotomous cut-off value of the PLR was identified to classify the patients into high and low PLR groups; (3) studies assessed the association of PLR with overall survival (OS), cancer-specific survival (CSS), and/or recurrence-free survival (RFS); (4) studies provided sufficient information for calculating hazard ratio (HR) and 95% confidence interval (CI); (5) published in English. The exclusion criteria were as follows: (1) case reports, conference abstracts, letters, editorials, reviews; (2) overlapping or duplicate studies; (3) irrelevant studies.

### Data Extraction and Quality Assessment

The selection of studies was conducted independently by two investigators (XW, XN) and any discrepancies were resolved by consensus. The following information were extracted from each study: first author, publication year, country, case number, age, cut-off values, study design, treatment method, and survival outcomes. The Newcastle-Ottawa Scale (NOS) was applied to evaluate the quality of all included studies (21). The NOS consisted of three parts: (a) selection (0–4 points); (b) comparability (0–2 points); and (c) outcome (0–3 points). The maximum score is 9 points and NOS scores ≥6 were assigned as high-quality studies.

### Statistical Analysis

Pooled HRs and 95% CIs were used to analyze the relationship between PLR and prognosis. Pooled odds ratios (ORs) and 95% CIs were used to analyze the relationship between PLR and clinicopathological features. Cochran's Q test and Higgins *I*^2^ statistic were used to assess the heterogeneity among studies. A *P* < 0.10 for *Q*-test or *I*^2^ > 50% for *I*^2^ test suggested significant heterogeneity and then a random-effect model (DerSimonian–Laird method) (22) was applied. Otherwise, the fixed-effect model (Mantel–Haenszel method) (23) was adopted. In addition, for the pooled HR estimates of OS and CSS, subgroup analysis by ethnicity, sample size, and cut-off value of PLR was conducted. Publication bias was estimated using Begg's funnel plot asymmetry tests (24). All analyses were carried out with the statistical software Stata, version 12.0 (Stata corporation, College Station, TX, USA). A two-sided *p* < 0.05 was considered statistically significant.

## Results

### Search Results and Study Characteristics

**Figure 1:**
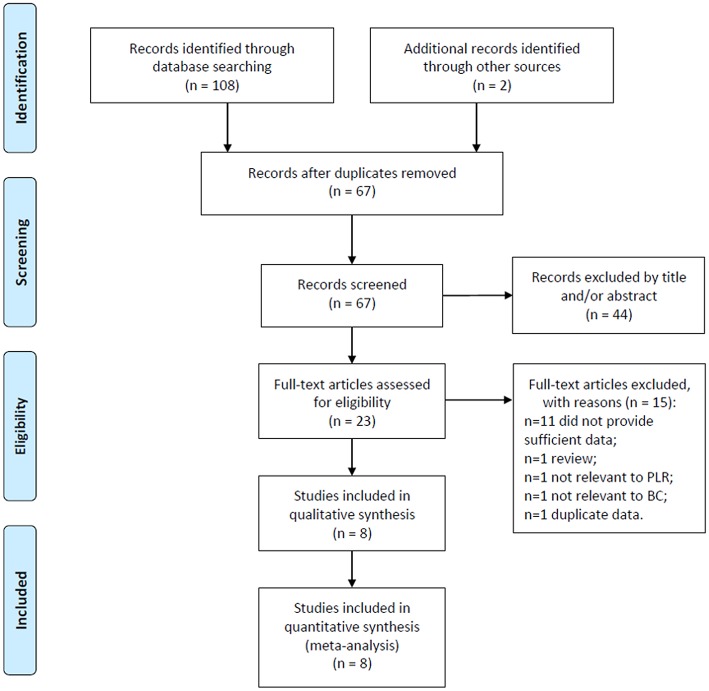
Flow chart of the included studies.

The flowchart of the literature selection process was shown in Figure 1. The initial retrieval of electronic databases identified 110 records; after duplicates were removed, 67 studies remained. After title and/or abstract examination, 44 papers were excluded and 23 records were evaluated by full-text reading. Fifteen full text studies were eliminated because of various reasons (Figure 1). Finally, 8 studies (15–19, 25–27) comprising a total of 3,303 patients, were included in this meta-analysis. The studies were published from 2015 to 2018 and were all retrospective studies. Of the 8 eligible articles, 3 studies (18, 25, 26) were from China, and 1 each from Canada (16), Korea (17), UK (15), Japan (19), and Poland (27), respectively. The cut-off values of PLR ranged from 123 to 218, with a median value of 150. All studies had a NOS score ≥6. The baseline characteristics of included studies were shown in Table 1.

**Table 1:** Baseline characteristics of studies included in the meta-analysis.

**References**	**Country**	**Dominant ethnicity**	**Sample size**	**Median/mean age (y)**	**Study design**	**Treatment**	**Cut-off value**	**Study period**	**Survival outcome**	**NOS score**
Bhindi et al. (16)	Canada	Caucasian	418	70	R	RC	150	1992–2012	OS, CSS, RFS	7
Kang et al. (17)	Korea	Asian	1551	65	R	TURB	124	1990–2013	OS, CSS	6
Lee et al. (15)	UK	Caucasian	226	75	R	TURB	218	2011–2013	OS	7
Mao et al. (18)	China	Asian	207	66	R	TURB	123	2010–2012	CSS, RFS	8
Miyake et al. (19)	Japan	Asian	117	72	R	RC	150	2006–2016	OS, CSS	7
Peng et al. (26)	China	Asian	516	66	R	RC	214	2006–2012	OS	8
Rajwa et al. (27)	Poland	Caucasian	144	NA	R	RC	160	2003–2015	OS, CSS	8
Zhang et al. (25)	China	Asian	124	65	R	RC	140	Jan–Dec, 2009	OS	6

*NA, not available; RC, radical cystectomy; TURB, transurethral resection of bladder tumor; OS, overall survival; CSS, cancer-specific survival; RFS, recurrence-free survival*.

### The Prognostic Value of PLR for OS

**Figure 2:**
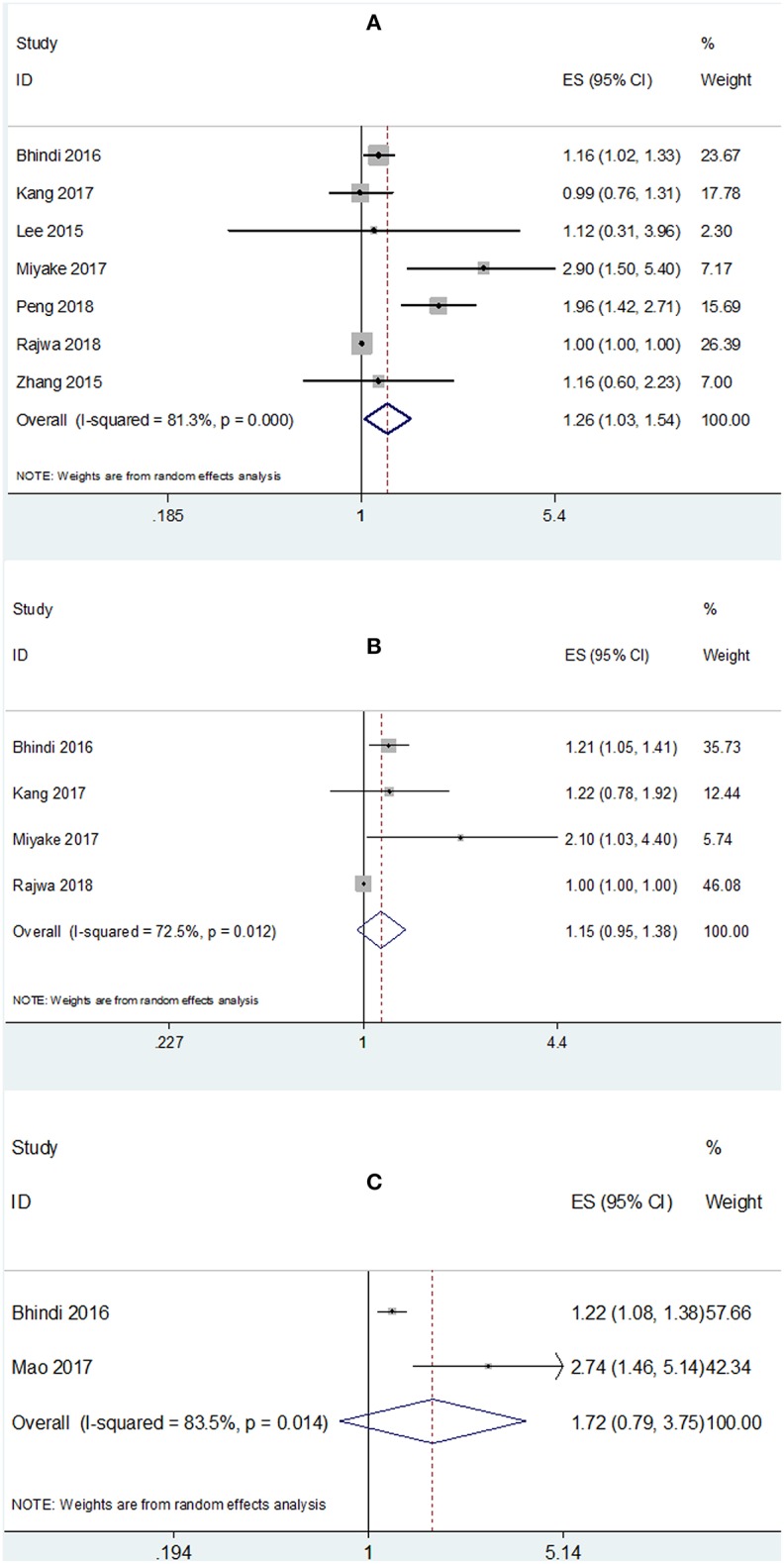
Meta-analysis of the association between PLR and **(A)** OS, **(B)** CSS, and **(C)** RFS mortality of bladder cancer.

There were 7 studies (15–17, 19, 25–27) providing data for estimating the association between PLR and OS in patients with bladder cancer. As shown in Figure 2A and Table 2, the pooled estimate of the high PLR for OS was significant, with the pooled HR being 1.26 (95% CI: 1.03–1.54, *p* = 0.026) with significant heterogeneity (*I*^2^ = 81.3% and *P* < 0.001). Subgroup analysis revealed that PLR has significant prognostic value for OS with cut-off value of PLR ≥ 150, whereas no significant prognostic significance was found regardless of ethnicity or sample (Table 2).

**Table 2:** Subgroup analysis of the association between PLR and OS, CSS, RFS.

**Survival analysis**	**No. of studies**	**HR (95% CI)**	***p***	***I*^**2**^ (%)**	***P*-value for heterogeneity**	**Analysis model**
**OS**						
Total	7	1.26 (1.03–1.54)	0.026	81.3	<0.001	Random
Ethnicity						
Asian	4	1.56 (0.96–2.54)	0.074	80.9	0.001	Random
Caucasian	3	1.06 (0.94–1.29)	0.369	57.5	0.095	Random
Sample size						
<200	3	1.42 (0.77–2.65)	0.264	72.9	0.011	Random
≥200	4	1.27 (0.95–1.72)	0.11	81.4	0.005	Random
Cut-off value						
<150	2	1.01 (0.79–1.3)	0.915	0	0.658	Fixed
≥150	5	1.37 (1.06–1.77)	0.017	87.5	<0.001	Random
**CSS**						
Total	4	1.15 (0.95–1.38)	0.149	72.5	0.012	Random
Ethnicity						
Asian	2	1.42 (0.97–2.08)	0.073	35.6	0.213	Fixed
Caucasian	2	1.09 (0.91–1.3)	0.377	83.9	0.013	Random
Sample size						
<200	2	1.32 (0.66–2.67)	0.434	74.9	0.046	Random
≥200	2	1.21 (1.05–1.39)	0.007	0	0.973	Fixed
Cut-off value						
<150	1	1.22 (0.78–1.91)	0.387	NA	NA	NA
≥150	3	1.14 (0.93–1.41)	0.213	80.4	0.006	Random
**RFS**						
Total	2	1.72 (0.79–3.75)	0.175	83.5	0.014	Random

*NA, not available*.

### The Prognostic Value of PLR for CSS

A total of 4 studies (16, 17, 19, 27) reported the data on PLR and CSS. The combined HR and 95% CI were: HR = 1.15, 95% CI = 0.95–1.38, *p* = 0.149 and the heterogeneity was significant (*I*^2^ = 72.5% and *P* = 0.012). Subgroup analysis demonstrated that PLR showed significant prognostic impact on CSS in studies with sample size ≥200 (HR = 1.21, 95% CI = 1.05–1.39, *p* = 0.007, Figure 2B and Table 2).

### The Prognostic Value of PLR for RFS

There were 2 studies (16, 18) presenting the data of PLR and RFS in bladder cancer. The pooled results indicated non-significant prognostic effect of PLR in RFS (HR = 1.72, 95% CI = 0.79–3.75, *p* = 0.175; *I*^2^ = 83.5%, *P* = 0.014; Figure 2C and Table 2).

### The Association of PLR and Clinicopathological Factors

**Figure 3:**
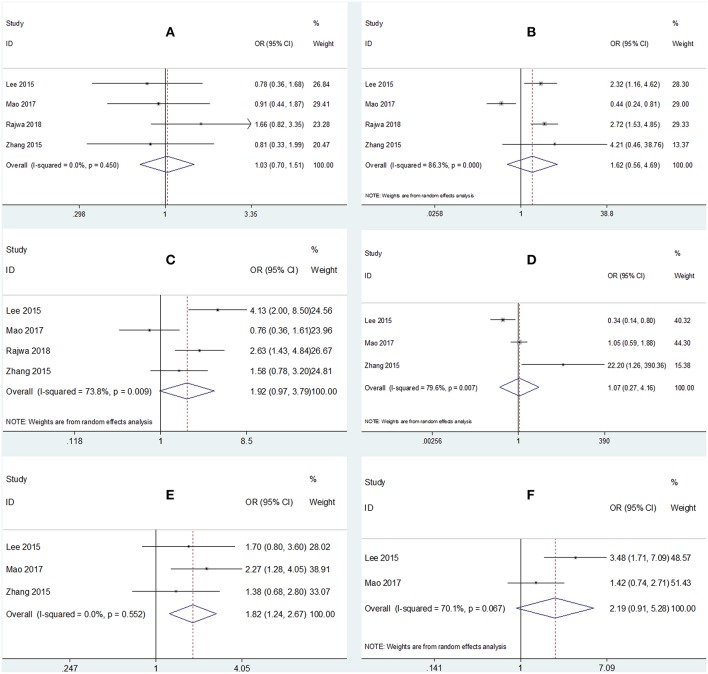
Forrest plots of associations between PLR and **(A)** sex, **(B)** tumor grade, **(C)** tumor stage, **(D)** distant metastasis, **(E)** age, and **(F)** tumor size.

Four studies (15, 18, 25, 27) reported the relationship between PLR and clinicopathological factors including sex, tumor grade, tumor stage, distant metastasis, age, and tumor size. As shown in Figure 3 and Table 3, high PLR was found to be significantly associated with age ≥ 65 years (OR = 1.82, 95% CI = 1.24–2.67, *p* = 0.002), whereas PLR was not significantly correlated with sex (OR = 1.03, 95% CI = 0.7–1.51, *p* = 0.884), tumor grade (OR = 1.62, 95% CI = 0.56–4.69, *p* = 0.373), tumor stage (OR = 1.92, 95% CI = 0.97–3.79, *p* = 0.06), distant metastasis (OR = 1.07, 95% CI = 0.27–4.16, *p* = 0.927), or tumor size (OR = 2.19, 95% CI = 0.91–5.28, *p* = 0.08).

**Table 3:** Meta-analysis results of PLR and clinicopathological parameters in patients with bladder cancer.

**Clinicopathological factors**	**No. of studies**	**OR (95% CI)**	***p***	***I*^**2**^ (%)**	***P*-value for heterogeneity**	**Analysis model**
Sex (M vs. F)	4	1.03 (0.7–1.51)	0.884	0	0.45	Fixed
Tumor grade (G3 vs. G1/G2)	4	1.62 (0.56–4.69)	0.373	86.3	<0.001	Random
Tumor stage (T2-T4 vs. Ta-T1)	4	1.92 (0.97–3.79)	0.06	73.8	0.009	Random
Distant metastasis (yes vs. no)	3	1.07 (0.27–4.16)	0.927	79.6	0.007	Random
Age (y) (≥65 vs. <65)	3	1.82 (1.24–2.67)	0.002	0	0.552	Fixed
Tumor size (cm) (≥3 vs. <3)	2	2.19 (0.91–5.28)	0.08	70.1	0.067	Random

### Publication Bias

**Figure 4:**
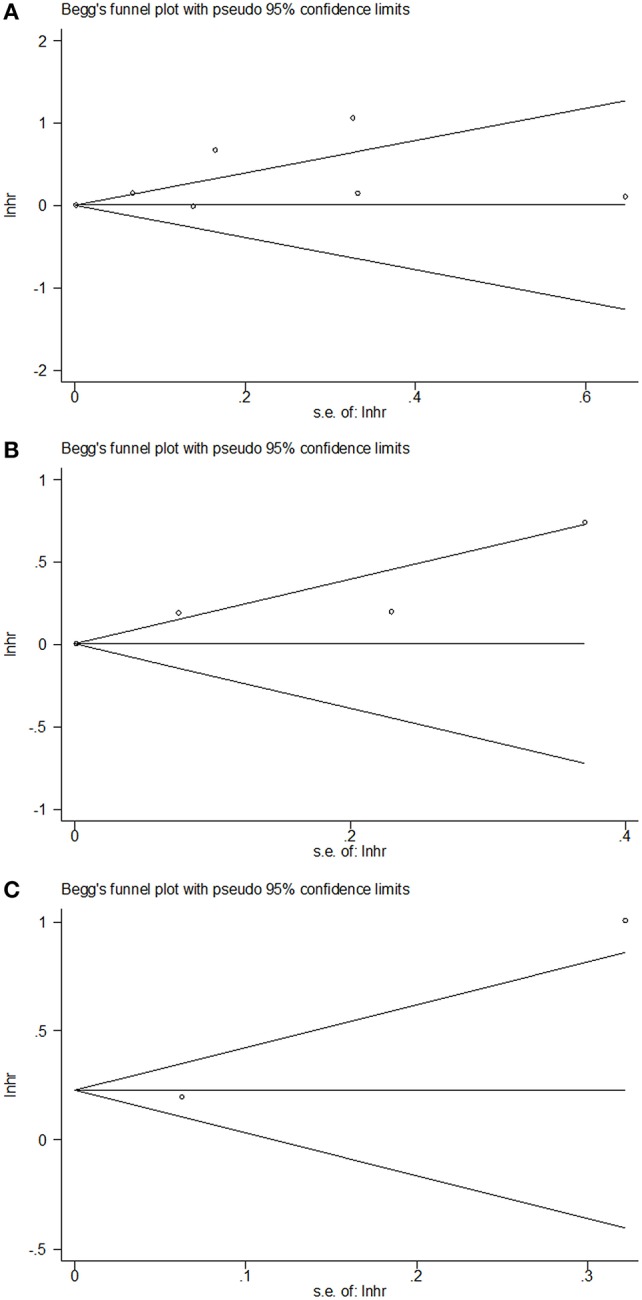
Begg's funnel plots of **(A)** OS, **(B)** CSS, and **(C)** RFS.

Publication bias was not significant in the current meta-analysis based on the plots of publication shown in Figure 4. The Begg's *p* for OS, CSS, and RFS were 0.764, 0.497, and 0.317, respectively.

## Discussion

In the present study, we comprehensively searched multiple databases and retrieved 8 studies including 3,303 patients with regard to the prognostic value of PLR for bladder cancer. To our knowledge, this study is the first meta-analysis to investigate the prognostic role and clinical relevance of PLR in patients with bladder cancer. The pooled data showed that a high PLR predicted poorer OS in bladder cancer, moreover, high PLR was correlated with patients aged 65 years and older. Taken together, this study indicated that the pretreatment PLR might be as a convenient and reliable biomarker in the prognosis of bladder cancer.

Recent studies have shown that the inflammatory nature of the tumor microenvironment plays a key role in tumor development, including initiation, growth, and metastasis (28, 29). It has been suggested that there is crosstalk between inflammatory responses and tumor progression (5). However, the potential mechanism of PLR affecting the survival of patients with bladder cancer is still largely unknown. This association may be explained by immune inflammation in the tumor microenvironment. Platelets can directly promote the growth of tumor cells by secreting vascular endothelial growth factor, basic fibroblast growth factor, platelet-derived growth factor and other angiogenesis and tumor growth factors (30–32). Platelet-derived micro vesicles can stimulate mitogen-activated protein kinases and promote tumor progression, metastasis, and angiogenesis in lung cancer cells (33). In addition, platelet-derived nucleotides can promote tumor-cell transendothelial migration and metastasis via P2Y2 receptor (34). In contrast, lymphocytes play an important role in T cell mediated antitumor response. Tumor infiltrating lymphocytes (TIL) are common inflammatory cells in the tumor environment and have been found to be involved in the anti-tumor immune response (35). In addition, high number of TILs have antitumor activity as judged by their favorable effect on cancer patients' survival (36). Therefore, the PLR combines the significance of platelet counts and lymphocyte counts and has the potential to be an effective prognostic biomarker.

Previous studies have shown the prognostic value of PLR in various cancers (7, 37). A meta-analysis including 11 studies showed that elevated PLR was associated with shorter OS (HR: 1.48, 95% CI: 1.24–1.76, *p* < 0.001) in patients with ovarian cancer (38). Li's work also demonstrated an elevated PLR was associated with unfavorable overall survival in patients with pancreatic cancer (39). Another recent study indicated that an elevated PLR was an effective prognostic marker of both OS (pooled HR = 2.10, 95% CI: 1.38–3.19, *p* = 0.001) and progression-free survival (PFS) (pooled HR = 3.45, 95% CI: 1.61–7.40, *p* = 0.001) in renal cell carcinoma (40). In the present meta-analysis, we found the prognostic role of PLR for poorer OS, which was in accordance with previous studies. Furthermore, we also identified the association of PLR and older age, which may emphases the prognostic value of PLR in aged bladder cancer patients.

This meta-analysis showed that high PLR predicted poor OS, but not CSS nor RFS. We think this finding may be correlated with the amount of included studies and duration of follow-up. First, 7 studies were included for OS analysis, whereas only 4 and 2 studies were included for CSS and RFS analysis. Because less studies were included, the pooled results may be at the risk of more bias, which is a possible reason for non-significant correlation of PLR and CSS or RFS. Second, we think it is more important, is the different duration of follow-up for OS, CSS, and RFS. For the same group of patients, the OS is regularly longer than CSS and RFS. A high PLR predicted poor OS, but not CSS nor RFS, which could be possibly explained by that PLR reflected the balance status of immune responses and cancer development. The clinical significance of PLR is emerging at a relatively long duration at course of disease, such as OS. Because the follow-ups of CSS and RFS is relatively short, so the prognostic value of PLR could be non-significant.

It is notable that the results showed that PLR was significantly associated with age ≥65 years (*p* = 0.002). Because as age advances, the immune system undergoes profound remodeling and decline (41). The immune responses in elderly patients were weaken compared to patients in middle age (41). We think this is the possible reason for the association between high PLR and old age. It is also possible that PLR and old age are cofound factors for poor OS. Actually, a high PLR was also associated with higher tumor stage (*p* = 0.06) and larger tumor size (*p* = 0.08), although they are not statistically significant. The correlation might be significant when more studies were included. Taken together, we think it is reasonable that PLR is associated with older age, but PLR might not simply be a surrogate for age. PLR is possible associated with other aggressive clinical factors when more studies were included. In the current meta-analysis, all included studies applied surgery for bladder cancer patients. Therefore, we recommend that PLR should be evaluated at diagnosis of MIBC, or before surgery. Patients with greater PLR (≥150) might be treated with more aggressive therapeutic strategies or at close follow-up after curative treatment. In addition, because all included studies used surgical resection, the impact of chemotherapy or radiation therapy could not be investigated in the current meta-analysis. Further studies on the impact of chemotherapy or radiotherapy on PLR are still needed.

Nevertheless, our study has several limitations. First, significant heterogeneity existed among studies. However, subgroup analyses showed that the heterogeneity disappeared in studies with PLR cut-off value <150 and studies with sample size ≥200. Second, the cut-off value of PLR applied in included studies was not uniform. Third, a large part of the included studies come from Asia. It is unclear whether these findings apply to other populations. Finally, all included studies were retrospective studies. Therefore, further large-scale prospective studies are needed to validate the results.

## Conclusion

Our results demonstrated that the pretreatment PLR was associated with worse OS in conjunction with older age clinicopathological features in patients with bladder cancer. Therefore, it is suggested that PLR is a promising biomarker for use in clinical management to predict survival outcome in bladder cancer.

## Author Contributions

GT collected and analyzed the data and wrote the paper. XW analyzed the data and participated in the writing of manuscript. XN assisted with the data analyses. XW and GT conceived and designed this study. All authors reviewed the paper, read, and approved the final manuscript.

### Conflict of Interest Statement

The authors declare that the research was conducted in the absence of any commercial or financial relationships that could be construed as a potential conflict of interest.
